# Quantifying floral shape variation in 3D using microcomputed tomography: a case study of a hybrid line between actinomorphic and zygomorphic flowers

**DOI:** 10.3389/fpls.2015.00724

**Published:** 2015-09-10

**Authors:** Chun-Neng Wang, Hao-Chun Hsu, Cheng-Chun Wang, Tzu-Kuei Lee, Yan-Fu Kuo

**Affiliations:** ^1^Institute of Ecology and Evolutionary Biology, National Taiwan UniversityTaipei, Taiwan; ^2^Department of Life Science, National Taiwan UniversityTaipei, Taiwan; ^3^Department of Bio-Industrial Mechatronics Engineering, National Taiwan UniversityTaipei, Taiwan

**Keywords:** three-dimensional image analysis, geometric morphometrics, petal shape, dorsoventral asymmetry, flower opening, floral morphology, *Sinningia speciosa*

## Abstract

The quantification of floral shape variations is difficult because flower structures are both diverse and complex. Traditionally, floral shape variations are quantified using the qualitative and linear measurements of two-dimensional (2D) images. The 2D images cannot adequately describe flower structures, and thus lead to unsatisfactory discrimination of the flower shape. This study aimed to acquire three-dimensional (3D) images by using microcomputed tomography (μCT) and to examine the floral shape variations by using geometric morphometrics (GM). To demonstrate the advantages of the 3D-μCT-GM approach, we applied the approach to a second-generation population of florist's gloxinia (*Sinningia speciosa*) crossed from parents of zygomorphic and actinomorphic flowers. The flowers in the population considerably vary in size and shape, thereby served as good materials to test the applicability of the proposed phenotyping approach. Procedures were developed to acquire 3D volumetric flower images using a μCT scanner, to segment the flower regions from the background, and to select homologous characteristic points (i.e., landmarks) from the flower images for the subsequent GM analysis. The procedures identified 95 landmarks for each flower and thus improved the capability of describing and illustrating the flower shapes, compared with typically lower number of landmarks in 2D analyses. The GM analysis demonstrated that flower opening and dorsoventral symmetry were the principal shape variations of the flowers. The degrees of flower opening and corolla asymmetry were then subsequently quantified directly from the 3D flower images. The 3D-μCT-GM approach revealed shape variations that could not be identified using typical 2D approaches and accurately quantified the flower traits that presented a challenge in 2D images. The approach opens new avenues to investigate floral shape variations.

## Introduction

Flowers are essential organs for reproduction in angiosperms. The flower shape can vary tremendously. The morphological variations in the corolla must be determined quantitatively to address questions regarding evolutionary divergence (Gómez et al., 2006; Feng et al., 2009), genotype-phenotype association (Cui et al., 2010; Hsu et al., 2015), plant-pollinator interactions (Yoshioka et al., 2005; Galliot et al., 2006; Gómez et al., 2008; van der Niet et al., 2010), and breeding selection (Yoshioka et al., 2006; Kobayashi et al., 2007; Kawabata et al., 2009; Kanaya et al., 2010). We proposed an approach to quantify the shape variations of corollas in three-dimensional (3D) images by using microcomputed tomography (μCT) and geometric morphometrics (GM).

The analysis of floral shape discrepancies is traditionally performed using classic morphometrics (Miller and Venable, 2003; Pérez et al., 2004; Kobayashi et al., 2007; Fernández-Mazuecos et al., 2013; Wessinger et al., 2014). Classic morphometrics uses multivariate statistics to measure distances between anatomical landmarks (i.e., characteristic points). The differences in distances between specimens are then evaluated. Shape is mathematically defined as the geometric information of an object except its scaling, translation, and rotation (Gower, 1975). Determining the distances between the landmarks neither reconstructs the original geometric relationship nor separates the shape information from the overall size of the specimens. Thus, the classic morphometrics approach has been considered less amenable in studies of floral shape variations (Dalayap et al., 2011; Fernández-Mazuecos et al., 2013).

Geometric morphometrics (Lawing and Polly, 2010; Zelditch et al., 2012) has been increasingly used to quantify the flower shape because of the recent advances in digital photography (Dalayap et al., 2011; Savriama et al., 2012). GM is a collection of algorithms that convey the spatial correlation on a set of landmarks identified from the photographic images of the objects to be analyzed. The method preserves the geometries of the landmark configurations. Thus, the statistical results of GM can describe the actual shape or form divergences. In recent years, numerous studies have applied curve-based GM techniques (Bo et al., 2014) for evaluating shape variations of individual petals (Yoshioka et al., 2005, 2007; Kawabata et al., 2009, 2011; Nii and Kawabata, 2011). Some other studies have used landmark-based GM techniques (Adams et al., 2004; Klingenberg, 2010) for examining the morphological divergence of corollas (Shipunov et al., 2004; Gómez et al., 2006, 2008; Feng et al., 2009; Kaczorowski et al., 2012; Savriama et al., 2012; Hsu et al., 2015).

Photographic images can adequately capture an object configuration only if the form of objects to be studied can be appropriately represented in 2D images. Corollas, however, have complex geometries. A considerable portion of information can be lost when corollas are depicted in 2D images. Thus, applying GM to landmarks identified from the 2D images of flowers can lead to suboptimal results for analyzing shape variations (Kuhl and Giardina, 1982). Therefore, authentic 3D images of the flowers must be acquired to retain the structural information implicit in corollas.

**Figure 1:**
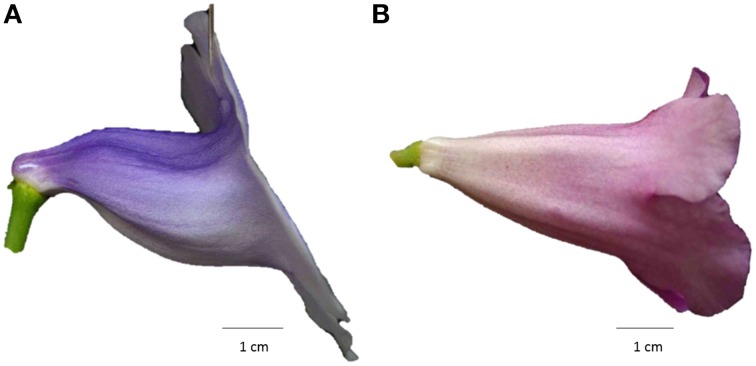
**Side-view images of (A) a trumpet-shaped zygomorphic flower and (B) a funnel-shaped actinomorphic flower from a hybrid line of ***S. speciosa*****.

Recent advances in modern scanning techniques make it feasible and affordable to reconstruct 3D images for objects. Typically, volumetric data on delicate botanical materials (e.g., flowers) are obtained using computed tomography or magnetic resonance image scanners. Furthermore, the high resolution of these technologies provides the detailed information required for accurately quantifying morphological variations. Studies have applied these 3D techniques to derive the shape equation for tomato (Li et al., 2011), perform vascular anatomy on living plants (McElrone et al., 2013), and visualize the structural changes occurring in plant leaves (Pajor et al., 2013). Particularly, van der Niet et al. (2010) suggested that combining 3D micro-computed tomography scanning with geometric morphometric methods could be a powerful strategy to accurately quantify patterns of floral shape variation. Motivated by their approach, we adopted a similar procedure to quantitatively evaluate corolla shape variations in 3D images by using μCT, GM, and image processing. The procedure was applied to the flowers of a second-generation (F_2_) population resulting from a crossing between a zygomorphic variety and an actinomorphic cultivar of florist's gloxinia (*Sinningia speciosa*; Hsu et al., 2015). These F_2_ flowers showed a considerable degree of variation in flower opening and corolla symmetry (Figure 1), thus serving as excellent materials for performing the quantification of floral shape variations.

The specific objectives of our study were to (1) develop tools for identifying flower regions in μCT images, (2) establish procedures for facilitating landmark selection, (3) identify the leading morphological variations of the flowers, (4) define and quantify physically measured traits such as flower opening and corolla asymmetry, (5) observe the floral shape transition between the zygomorphic and actinomorphic flowers, and (6) compare the floral shape analysis results obtained using 3D images with those obtained using 2D images.

## Materials and methods

### Flower materials

**Figure 2:**
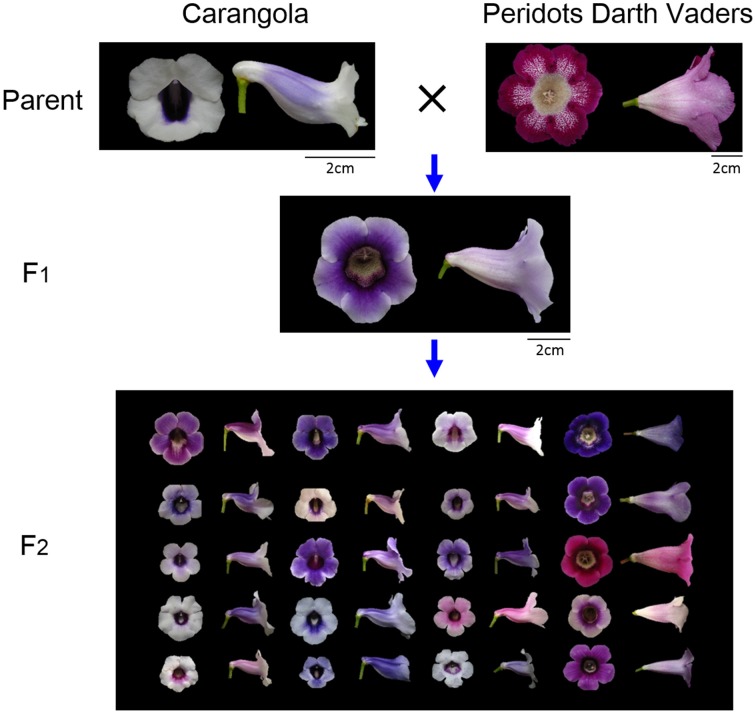
**Crossing process of ***S. speciosa*** flowers**. The parents were a zygomorphic variety “Carangola” and an actinomorphic cultivar “Peridots Darth Vaders.” The images of the F_2_ flowers were randomly selected from the 57 plants used in this study.

The flower samples were obtained by crossing two cultivars of *S. speciosa*, “Carangola” and “Peridots Darth Vaders” (Figure 2). These parental accessions were crossed to breed F_1_ plants. A total of 320 F_2_ plants were generated by selfing a single F_1_ plant. All the plants were grown in a greenhouse under natural lighting with 20% shade and 70–80% humidity at 22–28°C. We included only the flowers of the F_2_ plants with exactly 5 petal lobes because the flowers with different numbers of petal lobes were incomparable in shape (i.e., nonhomologous; Adams et al., 2004) and thus should be excluded from comparison.

### Flower image acquisition

**Figure 3:**
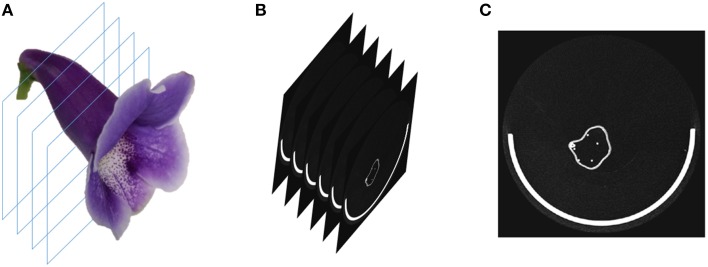
**(A)** Illustration of the μCT image scanning, **(B)** image slices from the scanning, and **(C)** a single raw image slice. The white semicircle at the bottom of the image slice is the base for fastening the flower. The raw image slice contained white sparkles (noise) in the background.

Three-dimensional flower images were acquired using a μCT scanner (SkyScan 1076, Bruker, Kontich, Belgium). The specimens of fully bloomed (complete anthesis) fresh flowers were cut at the pedicel near the bottom of the tube and placed in the scanner chamber. The specimens were fastened to the base in the chamber with gummed tape to prevent the movement of the specimens during scanning. The transverse diameter of the chamber was 68 mm and the single scan length was 20 mm in the travel direction of the scanner (i.e., the direction perpendicular to the transverse plane; Figure 3A). The number of scans was dependent on the flower sizes. The X-ray source voltage, current, exposure time, and scanning resolution were set to 40 kV, 250 μA, 150 ms, and 35 μm, respectively. The scanning resolution was identical along the X, Y, and Z-axis. After scanning was completed, the 3D raw images were reconstructed by using SkyScan NRecon (Bruker, Kontich, Belgium). We acquired 57 flower images, each of which was from an F_2_ individual. The acquisitions were performed between August, 2012 and September, 2014. The image sizes ranged from 5.0 to 9.3 GB.

### Flower region segmentation

The raw images comprised flower specimens and the base for fastening the flower samples. Image processing algorithms were applied to segment the flowers from the background, reduce the noise of the images, and transform the images into an appropriate format for the subsequent analysis. These algorithms were implemented in a graphical user interface program developed using MATLAB (The Mathworks, Natick, MA, USA; see Supplementary Presentation 1, Supplementary Datasheet 1, and Supplementary Video 1) and were performed automatically. Before the processing, the spatial resolution of the raw images was reduced by 50% to a voxel size of 70 μm on each side. This resolution was chosen to expedite the processing, while the details of the flowers were still available.

**Figure 4:**
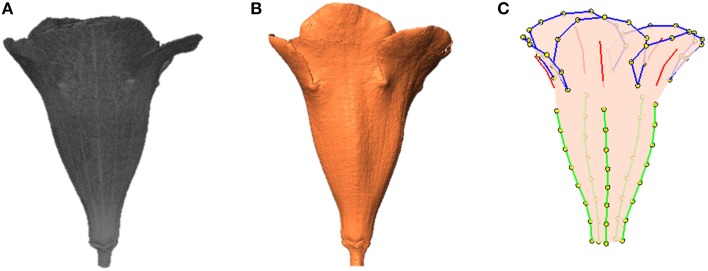
**(A)** Volumetric image, **(B)** surface image, and **(C)** landmarks of a flower. Green lines, tube midribs; red lines, lobe midribs; blue lines, lobe contours; yellow dots, landmarks.

To perform the processing, the operator selected a folder containing the raw image of a flower. The raw image comprised 2D grayscale slices (e.g., images of the transverse plane; Figures 3B,C) collected along the travel direction. The algorithms were applied on a slice at a time. First, the base, typically the greatest object located at a fixed position in the slice, was recognized and eliminated using a series of operations. Next, the contrast (i.e., gamma value) of the slice was adjusted appropriately to span the grayscale dynamic range. The slice was then binarized. Connected-component labeling (Haralock and Shapiro, 1991) was next employed to detect objects in the slice. The objects with pixel sizes smaller than a certain threshold (i.e., sparkles) were regarded as noise and were removed. The resulting slice served as an image mask. Subsequently, the original grayscale slice was masked (Gonzalez and Woods, 2006) using the image mask to retain the region of interest. A morphological closing (Vincent, 1994) was next adopted to eliminate the hollow pixels within the flower petals. The structuring element for the closing operation was a disc with a radius of 1 pixel. The aforementioned operations were proceeded until all the slices were processed. The collection of the slices, referred to as a volumetric image (Figure 4A), was then converted to a surface image (Figure 4B). The surface image (Hansen and Johnson, 2005) comprised of fine triangle meshes covering the surface of the flower. The mesh density was adjusted to maintain a reasonable resolution of the image. The surface image was stored in polygon file format (i.e., a PLY file) for the subsequent analysis.

### Landmark identification

**Figure 5:**
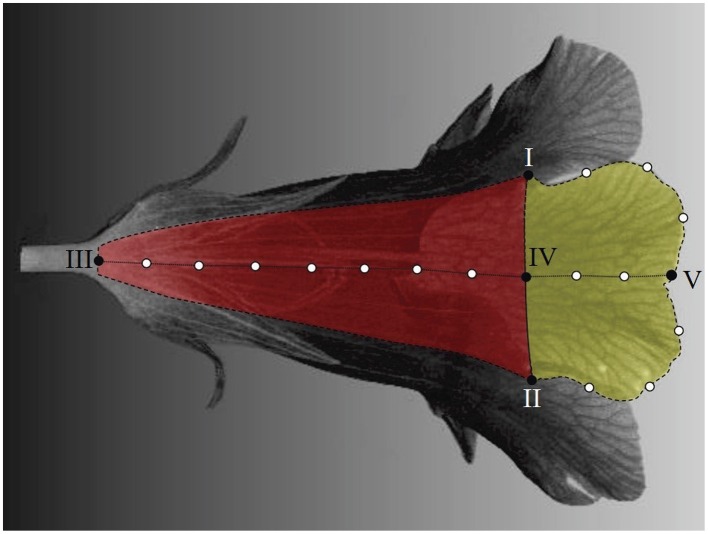
**Primary and secondary landmarks on a petal**. Solid dots, primary landmarks; hollow dots, secondary landmarks; red area, tube compartment; yellow area, lobe compartment; dotted line, petal midrib.

Landmarks are categorized as primary and secondary (Zelditch et al., 2012). In this study, the primary landmarks were defined as the anatomically recognizable points of the corolla, including the intersections of adjacent lobes (landmarks I and II in Figure 5), proximal and distal points of petal midribs (landmarks III and V), and boundary points of lobes and tubes on petal midribs (landmark IV). The secondary landmarks were equally distributed points between two primary landmarks along the lobe contours or petal midribs (hollow dots in Figure 5).

The landmarks were identified semi-automatically. The identification was performed for a petal at a time. The process involved two phases—a manual selection and an automatic determination. The manual selection was performed using Landmark software (Wiley et al., 2005). First, the operator manually selected the lobe intersections and midrib proximal point (landmarks I, II, and III in Figure 5). The boundary point of the lobe and tube on the midrib (landmark IV) was then determined as the surface point of the petal that is associated with the shortest Euclidean distance to the middle point between landmarks I and II. Next, the operator manually determined the lobe contour and petal midrib. To do so, approximately 70 and 80 points, respectively, were selected along the lobe contour and petal midrib. The points were selected with the best effort to be evenly distributed on the lobe contour and petal midrib. These points were then stored in a consecutive order for the subsequent landmark determination.

The midrib distal point (landmark V) and secondary landmarks were determined automatically. The automatic landmark determination was performed using a program developed using MATLAB (see Supplementary Presentation 2, Supplementary Datasheet 1, and Supplementary Video 2). The program read the selected points and modeled the lobe contour and petal midrib using piecewise linear interpolation between consecutive points. The equidistant point from landmarks I and II in the geodesic space (along the lobe contour) was determined as the midrib distal point (landmark V). The program then determined the secondary landmarks as the equally distributed points along the lobe contour and petal midrib. In this study, 3, 2, and 7 secondary landmarks, respectively, were identified on the semi-lobe contour (the dashed lines connecting I–V and II–V), lobe midrib (the dotted line connecting IV–V), and tube midrib (the dotted line connecting III–IV). These numbers were chosen to adequately illustrate the flower shape and to balance the numbers of the landmarks on the lobe and tube.

As a result, a total of 95 landmarks, including 20 primary and 75 secondary, were collected for each flower (Figure 4C). The lobes and tubes comprised 55 and 50 landmarks, respectively, with 10 landmarks in common. *S. speciosa* and many other species in the Lamiales natively develop flowers with limited anatomical points that can serve as the primary landmarks. The proposed approach for selecting the secondary landmarks in 3D images effectively increases the number of homologous characteristic points of the flowers being studies, thus improving the overall quality and capability of describing and illustrating the flower shapes.

### Identification of major shape variation

GM was applied to the landmarks for identifying the major shape variations among the flowers. The GM procedure includes generalized Procrustes analysis (GPA; Gower, 1975; Rohlf and Slice, 1990) and principal component analysis (PCA; Jolliffe, 2002). GPA was performed to remove the variation irrelevant to shape (e.g., translation, scaling, and rotation). In the GPA analysis, the mean geometric center of all the flowers was calculated. The geometric center of each flower was translocated to the mean. Next, the mean landmark coordinates of all the flowers were calculated. Scaling and rotation operations were applied to each flower for minimizing the sum of squared distances between the landmarks of the flower and the mean landmarks. The process was performed iteratively until no further reduction could be achieved in the sum of squared distances. The resulting landmarks from the GPA analysis were then subject to PCA. PCA identified shape variations and their corresponding principal components (PCs). The PCs were sorted in descending order by the percentage of the variance between the flowers. The first few PCs accounted for a large proportion of the variance and could represent the major shape variation among the flowers. The floral shape variation could also be visualized by reconstructing the flowers using inverse PCA with altered PC values.

### Morphological traits: Flower opening and corolla asymmetry

**Figure 6:**
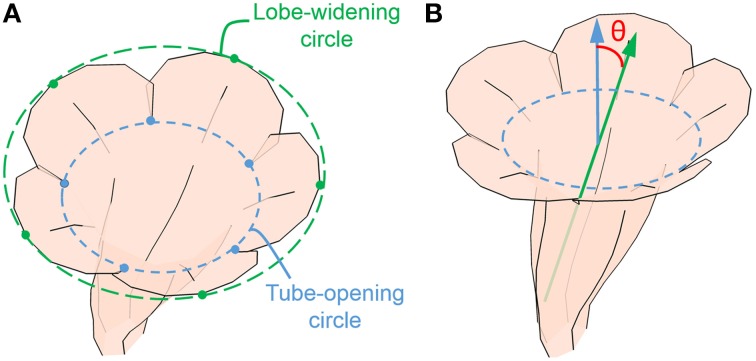
**(A)** Tube-opening and lobe-widening circles for calculating the opening score and **(B)** asymmetry angle. Green arrow, the long axis of corolla; blue arrow, the normal vector of tube-opening circle.

The GM analysis revealed that flower opening (i.e., corolla curvature) and dorsoventral symmetry were the leading shape variations. Two traits, flower opening and corolla asymmetry, were defined and directly assessed in the 3D flower images using image processing and computer graphics techniques. Flower opening was defined as the ratio of the diameter of lobe-widening circle to the diameter of tube-opening circle (Figure 6A). The lobe-widening circle was defined as the circle that optimally fitted the 5 petal midrib distal points (landmark V in Figure 5). The tube-opening circle was defined as the circle that optimally fitted the 5 lobe intersections. Corolla asymmetry was defined as the sine value of the asymmetry angle. The asymmetry angle (θ in Figure 6B) was the angle between the long axis of the corolla tube and the normal vector of the tube-opening circle. The long axis of the corolla tube was the first principal axis of the positions of the tube landmarks, and it was obtained using PCA. On the basis of these definitions, the two traits were unaffected by the size, translation, or rotation of the 3D flower images.

## Results

### Three-dimensional flower images and landmarks

**Figure 7:**
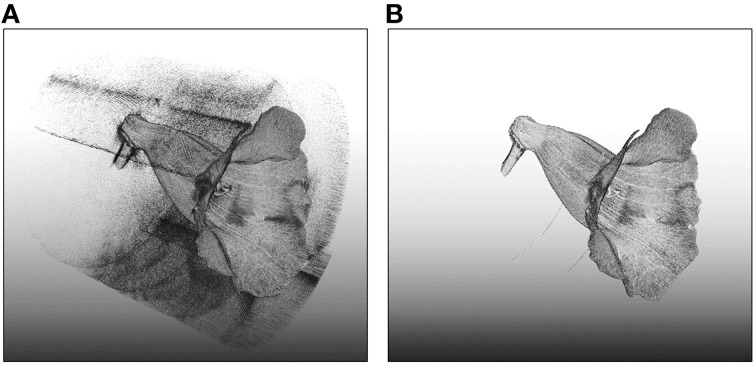
**Volumetric images of a flower (A) before and (B) after the noise reduction**. The image before noise reduction contained considerable amount of sparkles (noise) in the background.

**Figure 8:**
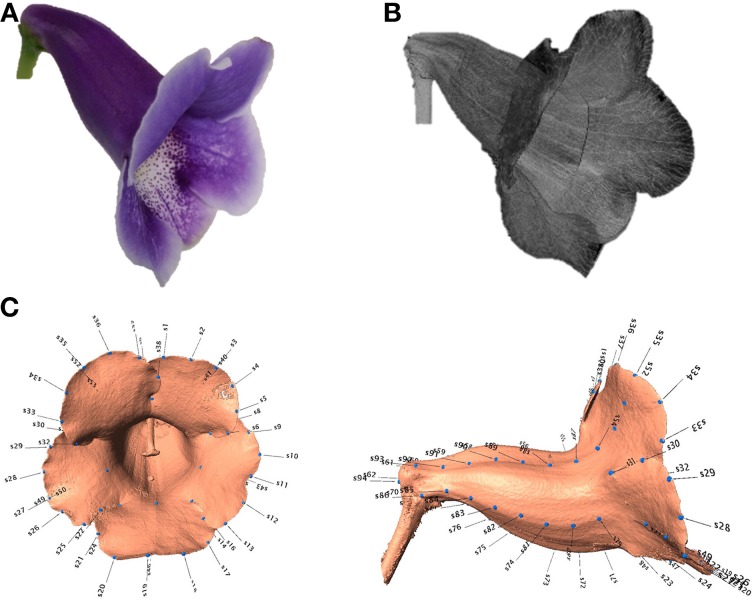
**(A)** Image of a flower, **(B)** corresponding μCT image, and **(C)** landmarks (blue dots with numbered labels) on the flower image.

Three-dimensional images of the flowers were acquired. Image processing algorithms were applied to segment the flower specimen from the background and to reduce noise in the images. Figure 7 illustrates the volumetric images of a flower before and after the noise reduction. In Figure 7, the base for fastening the flower was removed for illustration purposes. The sparkles in the background were considerably diminished after the noise reduction. Figures 8A,B show a flower image and its corresponding volumetric image. Landmarks were selected following the proposed procedure. Figure 8C shows the landmarks and their identification numbers.

### Identification and visualization of floral shape variations

PCs describing the primary floral shape variations were derived. The first three PC scores, PC1, PC2, and PC3, accounted for 38.8, 16.3, and 5.6% of the total shape variation. Each of the remaining PC scores accounted for less than 4% of the total shape variation. Because the first three PCs accumulated more than 60% of the total shape variation, we presented the results of the first three PCs only. The PCs were uncorrelated and normally distributed (see Supplementary Datasheet 1).

**Figure 9:**
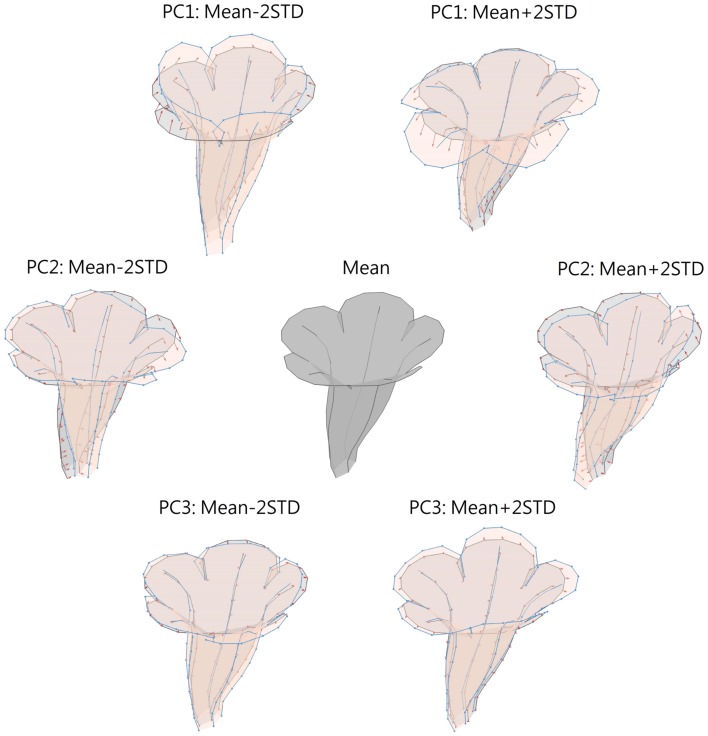
**Illustration of flower shape variations caused by changes in PCs**. Gray, mean flower shape; beige, reconstructed flowers with manipulated PCs; red arrows, direction and degree of transformation from the mean shape to another.

**Figure 10:**
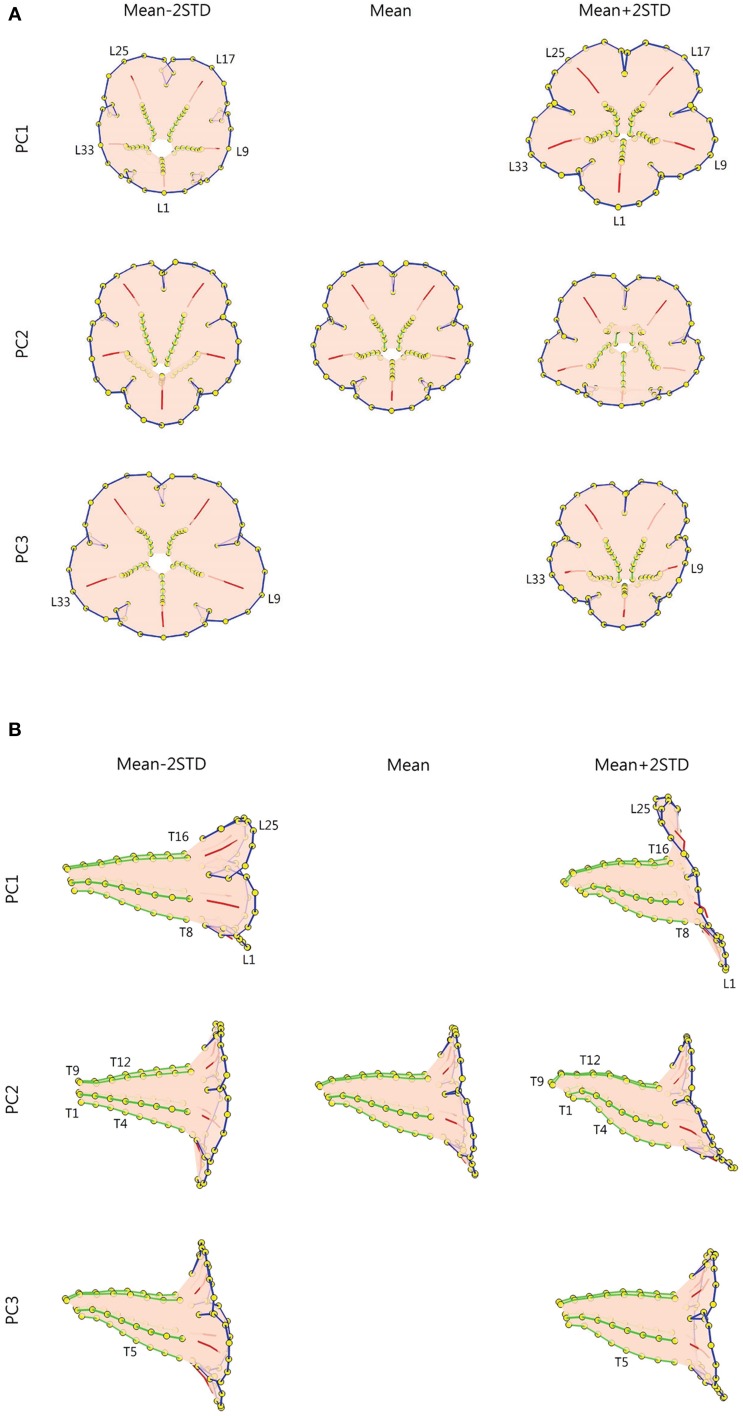
**(A)** Front-view and **(B)** side-view illustration of the flower shape variations. Green lines, tube midribs; red lines, lobe midribs; blue lines, lobe contours; yellow dots, landmarks.

Figure 9 illustrates the degree of floral shape variations caused by changes in the PCs. In the visualization process, the mean, and standard deviation (STD) of the PCs were calculated. Reconstructed landmarks were calculated using an inverse PCA with a specific PC value being manipulated, whereas other PC values were maintained at the mean values. The manipulated PC values were set at the mean or mean ± 2 STD. Flower shapes were then reconstructed using the resulting landmarks. The flowers were illustrated in 3D to reveal the degree of shape transformation. In Figure 9, the mean flower shape is indicated in gray, and the reconstructed flowers with the manipulated PCs are illustrated in beige. Red arrows at the landmarks show the direction and degree of transformation from the mean shape to another. Major transformation was observed at the distal lobes (PC1 and PC2), boundary between the lobe and tube (PC2), margin between the tube and sepal (PC2), and tube chamber (PC3). Figure 10 shows the front (or face) and side views of the flowers.

The shape variation associated with each PC was examined. We observed that PC1 primarily corresponded to corolla curvature and flower opening. Figures 9, 10 indicate that petal curvature in the boundary region between the lobe and tube changes drastically for the flowers with different PC1 values. The lobes of the flower with a high PC1 value bent outward at a considerable degree (the curves connecting L1–T8 and L25–T16 in Figure 10B). This large curvature produced a wide opening in the flower. The landmarks on lobe contours (from L1 to L33 in Figure 10A) spread out from the center. By contrast, the flower with a low PC1 value was associated with a moderate degree of opening (Figure 10B). In the front-view images, the lobes of the flower with a narrow opening (mean – 2 STD) exhibited a high degree of overlapping compared with the lobes of the flower with a wide opening (mean + 2 STD) in which the lobes were distinctly separated.

We observed that PC2 mainly corresponded to the degree of corolla dorsoventral symmetry. The flower with a low PC2 value was actinomorphic. The distances from either side of the petal base (the lines connecting T1–T4 and T9–T12 in Figure 10B) to the center of the tube were balanced. By contrast, the flower with a high PC2 value was zygomorphic. The end of the tube (the lines connecting T1–T4 and T9–T12) bent asymmetrically upward, resulting in a tube length difference between the dorsal and ventral petals. In addition, PC2 corresponded to the degree of overlapping between the ventral and lateral lobes in the front view (Figure 10A). Compared with the actinomorphic flower, the zygomorphic flower developed a ventral lobe bent downward at a higher degree (Figure 10B). Because of the aforementioned changes, the flowers with various PC2 values displayed distinct front views (Figure 10A) for pollinators.

We observed that PC3 particularly corresponded to the size of the tube chamber. The flower with a high PC3 value was associated with a chamber more dilated around landmark T5 (Figure 10B). Furthermore, the flower with a small PC3 value was associated with widely opened lateral lobes (L9 and L33 in Figure 10A).

### Variation in morphological traits

**Figure 11:**
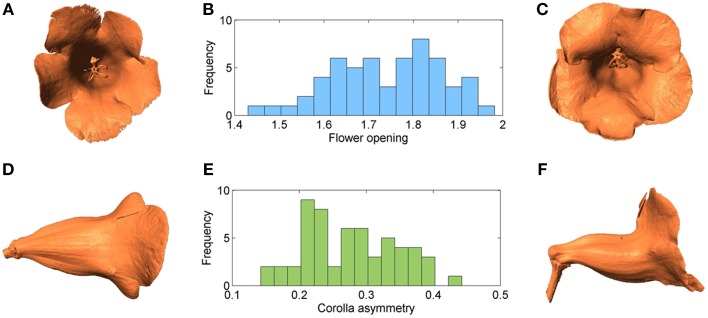
**(A)** Flower associated with the smallest opening value, **(B)** histogram of flower opening, **(C)** flower associated with the largest opening value, **(D)** flower associated with the smallest corolla asymmetry value, **(E)** histogram of corolla asymmetry, and **(F)** flower associated with the largest corolla asymmetry value.

Figure 11 shows the distributions of flower opening and corolla asymmetry. The mean and STD of flower opening were 1.74 and 0.12, respectively. The mean and STD of corolla asymmetry were 0.28 and 0.07 (the corresponding asymmetry angles were 16.19° and 4.19°), respectively. Figures 11A,C show the images of the flowers with the extreme flower opening values (1.43 and 1.98). Figures 11D,F show the images of the flowers with the extreme corolla asymmetry values (0.14 and 0.44).

The two traits could be unimodally and continuously distributed (Figure 11). The hypothesis that flower opening and corolla asymmetry were normally distributed could not be rejected by the Kolmogorov-Smirnov test (*P* = 0.45 and 0.49). These observations suggested that a polygenic basis of these traits existed. In addition, the correlation coefficient between the two traits was 0.195, indicating that the development of these traits were likely independent.

### Comparison of shape variation analyses performed using 2D and 3D images

**Figure 12:**
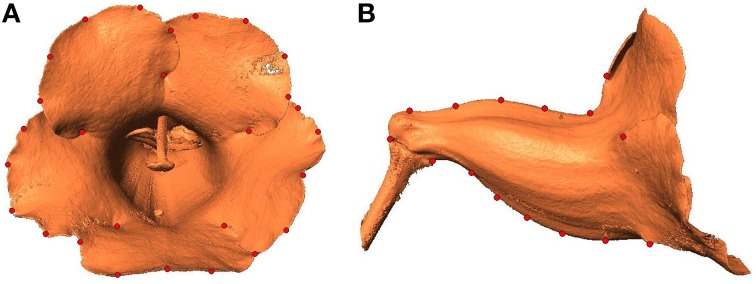
**2D (A) front-view and (B) side-view images of a flower**. Red dots, landmarks selected along the contours.

The performance of the proposed approach was compared with that of the conventional method, which determines the floral shape variations by using 2D images (Hsu et al., 2015). The 2D images were obtained by projecting the 3D flower images onto 2D planes. In the projection, the view angle of a flower was set according to its tube-opening circle (Figure 6A) and dorsoventral planes to capture the front-view and side-view images of the flower (Figure 12). This projection process mimicked the action of acquiring 2D flower images by using a camera. Subsequently, landmarks were identified on the images by following the procedure stated in a previous study (Hsu et al., 2015). All the front-view landmarks were located on the lobe contours, whereas all the side-view landmarks were located on the tube contours (Figure 12). This limitation was because of the challenge of accurately determining landmarks on the tubes from front views and on the lobes from side views. Thus, 30 front-view and 15 side-view landmarks were collected for each specimen (Figure 12).

**Figure 13:**
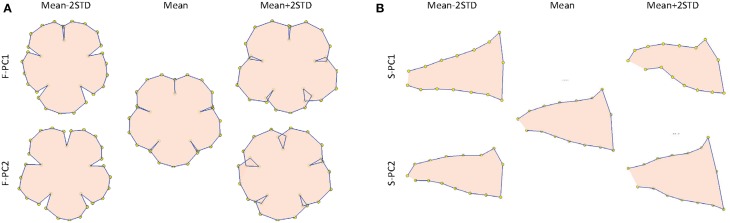
**(A)** Front-view and **(B)** side-view illustrations of the floral shape variation quantified using 2D images. The front-view and side-view illustrations only describe the shape changes in the lobe and tube contours, respectively.

To quantify the floral shape variation, two GM analyses were conducted using the front-view and side-view landmarks, respectively. This procedure followed the typical approach used for 2D images (Kawabata et al., 2009; Hsu et al., 2015). The first two PCs obtained from the front-view landmarks, referred to as F-PC1 and F-PC2, accounted for 19.0 and 14.5% of the total shape variation. The first two PCs obtained from the side-view landmarks, referred to as S-PC1 and S-PC2, accounted for 44.0 and 16.2% of the total shape variation. Figure 13 displays the floral shape variation caused by the changes in the first two PCs. We observed that F-PC1 and F-PC2 primarily corresponded to the ventral lobe extension and the degree of overlapping between the lobes. Furthermore, S-PC1 and S-PC2 principally corresponded to the dorsoventral asymmetry and the opening of the tube chamber. The flower opening (i.e., corolla curvature) characteristic shown in the 3D GM analysis was not observed in the 2D GM analysis.

## Discussion

### Advantages of 3D floral shape analysis

The 3D analysis explored the additional aspects of the corolla shape variation that was not observed using the conventional 2D methods. Our proposed approach can identify corolla curvature (i.e., flower opening). The 3D GM analysis revealed that the corolla curvature corresponded to the major portion of the total shape variation (i.e., PC1). However, this was not identified by the 2D GM analysis (Figure 13). Corolla curvature has been demonstrated to act as a mechanical nectar guide, which facilitates direct flower handling for plant-pollinator interactions (Campos et al., 2014). The corolla curvature is perhaps an essential trait for the development and evolution of flower shape.

Three-dimensional images enables quantification of flower traits. Flower shape is complex, and the principal shape variations are often presented qualitatively (e.g., GM analysis results). By using 3D images, flower traits corresponding to the leading shape variations can be further defined and measured with high accuracy. In this study, the traits of the corolla, such as the tube-opening circle, lobe-widening circle, and long axis, were quantified. Subsequently, the flower opening and corolla asymmetry scores were derived. These traits of flowers are physically measured and can quantitatively represent the flower shapes. Furthermore, these traits are crucial parameters that illustrate the transition between the zygomorphic and actinomorphic flowers. By contrast, these flower traits could be difficult to assess or quantify with a high level of uncertainties when 2D images are used. These traits can be used in future studies that address topics such as genotype-phenotype association or plant-pollinator interactions.

Graphics using 3D information are more powerful tools that illustrate flower shapes. With 3D coordinates, the corollas could be observed from various view angles in more detail (e.g., midribs). In addition, the lobe and tube of a corolla could be illustrated together in a 3D image (Figures 9, 10), whereas a 2D image could only demonstrate the lobe or tube of a corolla separately (Figure 13). The partial information obtained in 2D images may result in the misinterpretation of the floral shape variations. For example, the 2D graphical illustration (Figure 13A) could lead to a false interpretation of the shape variation corresponding to F-PC1 as the degree of overlapping for the ventral lobe, whereas the same shape variation was clearly observed as dorsoventral asymmetry in the 3D graphical illustration (Figures 9, 10).

### Reasons for 3D analysis than a 2D analysis

Three-dimensional images inherently contain more anatomical details (e.g., midribs). Landmarks must be situated on the homologous loci in all specimens and are typically identified on the basis of these anatomical details. By contrast, a large portion of geometric details are not available in 2D images. Thus, less shape variations can be quantified using 2D images. In addition, certain 2D landmarks are identified on the lobe or tube contours (Figure 12). These contours are projections on 2D planes and are subjected to the view angle of a camera. Thus, uncertainties can be introduced in the contours when the flower images are taken. Subsequently, these uncertainties propagate to the landmark coordinates. Moreover, a 3D flower image comprises both the lobe and tube landmarks of the same flower. The lobe and tube landmarks are subjected to the GM analysis simultaneously; therefore, the association between the two compartments can be retained. However, a 2D image comprises only the lobe or tube landmarks (Figure 13). Conducting the shape analysis by using only one of the datasets separately leads to a loss of association between the two compartments, therefore failing to retain the inherent shape information.

### Biological implications of flower shape variations

Our 3D GM analysis facilitated in identifying the flower opening and corolla asymmetry (indicated by the asymmetry angle) as the two major traits for the petal shape variations in the transition between actinomorphic and zygomorphic flowers. Wide flower opening and bilateral symmetry in the zygomorphic F_2_ individuals could attract pollinators and allow only those that enter flowers in a certain direction, thus facilitating pollen deposition on these visitors. Narrow flower opening and radial symmetry in the actinomorphic F_2_ individuals indicates that the flowers are unable to restrict pollinators entering from any direction. Flowering plants with bilateral symmetry have been demonstrated greatly in facilitating plant-pollinator interactions or coevolution (Citerne et al., 2010). We also demonstrated that an increased degree of flower opening is apparently associated with corolla asymmetry and together they establish the zygomorphic structure of the flower.

## Concluding remarks

The present study proposed approaches to facilitate the quantification of floral shape variations in 3D using μCT, image processing, and GM. A software was developed to reduce noise in 3D images and to segment flowers from the background automatically. Another software was developed to assist landmark determination semi-automatically. These tools expedite the processing of complex 3D images and enabled the selection of 95 landmarks on a flower. The procedures were applied to an F_2_ population crossed from two cultivars of *S. speciosa* with flowers in actinomorphic and zygomorphic forms. Three-dimensional images acquired using a μCT determined the floral shape variations as a whole and measured the morphological traits accurately (70 μm/voxel). The proposed 3D-μCT-GM approach revealed shape variations that could not be identified using typical 2D approaches and accurately quantified the flower traits that presented a challenge in 2D images. This approach has potential for application in future studies on genotype-phenotype associations or evolutionary divergence.

## Author contributions

The flower material was prepared by HH and CNW. The experiments were conceived and designed by YK. The experiments were performed by TL and CCW. The data were processed, analyzed, and interpreted by TL, CCW, HH, and YK. The manuscript was prepared by CCW, HH, CNW, and YK.

## Funding

This research was supported by the National Science Council (Ministry of Science and Technology) of Taiwan, grant NSC-101-2313-B-002-050-MY3 to YK and NSC-95-2311-B-002-014-MY3 to CNW.

### Conflict of interest statement

The authors declare that the research was conducted in the absence of any commercial or financial relationships that could be construed as a potential conflict of interest.
