# Supplementary figures and images for: Quantifying floral shape variation in 3D using microcomputed tomography: a case study of a hybrid line between actinomorphic and zygomorphic flowers
Source: Front Plant Sci. 2015 Sep 10;6:724. doi: 10.3389/fpls.2015.00724 (PMC4564768; doi:10.3389/fpls.2015.00724)

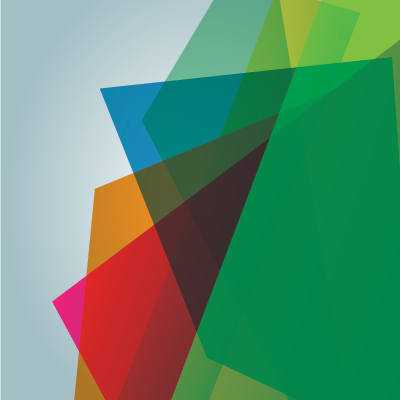

Supplement: Supplementary file 3 [file Presentation1.ZIP › Supplement1/Program1-ROI/splash.png]
