# Supplementary material for: Quantifying floral shape variation in 3D using microcomputed tomography: a case study of a hybrid line between actinomorphic and zygomorphic flowers
Source: Front Plant Sci. 2015 Sep 10;6:724. doi: 10.3389/fpls.2015.00724 (PMC4564768; doi:10.3389/fpls.2015.00724)
Supplement: Supplementary file 5 [file DataSheet1.DOCX]

***Supplementary Material***

**Quantifying floral shape variation in 3D using microcomputed tomography: a case study of a hybrid line between actinomorphic and zygomorphic flowers**

**Chun-Neng Wang** **^1,2^, Hao-Chun Hsu^2†^, Cheng-Chun Wang^3^, Tzu-Kuei Lee^3^, Yan-Fu Kuo^3^***

^1^Institute of Ecology and Evolutionary Biology, National Taiwan University, Taipei, Taiwan

^2^Department of Life Science, National Taiwan University, Taipei, Taiwan

^3^Department of Bio-Industrial Mechatronics Engineering, National Taiwan University, Taipei, Taiwan

**^†^**These authors contributed equally to this study.

*** Correspondence:** Dr. Yan-Fu Kuo, Department of Bio-Industrial Mechatronics Engineering, National Taiwan University, No. 1, Sec. 4, Roosevelt Rd. Taipei, 106, Taiwan. ykuo@ntu.edu.tw

1. **Flower segmentation and image quality improvement**

Figure S1 displays the screenshot of the flower segmentation and quality improvement program. Please find demonstration video in the supplement file for the detailed operation procedure.

1. **Landmark identification**

Figure S2 displays the screenshot of the landmark identification program. Please find demonstration video in the supplement file for the detailed operation procedure.

1. **Distributions of principal components**

Figure S3 displays the scatter plots of PCs. The PCs were standardized with zero means and unit variances. Unimodal distributions were observed for the first three PCs. Kolmogorov–Smirnov test indicated that the hypothesis that PC1, PC2, and PC3 were normally distributed could not be rejected (*P* = 0.94, 0.84, and 0.96).

1. **Tube-opening circle, lobe-widening circle, and asymmetry angle**

Figure S4 displays the boxplots of diameters for the tube-opening circle and lobe-widening circle. The mean diameters of the tube-opening circle and lobe-widening circle were 2.7 and 4.78 cm, respectively. The mean of the corolla asymmetry angle was 16.19°.

**Figure legends**

**Supplementary figure 1:** The screenshot of the flower segmentation and quality improvement program.

**Supplementary figure 2:** The screenshot of the landmark identification program.

**Supplementary figure 3:** Scatter plots of the first three principal components.

**Supplementary figure 4:** **(A)** Boxplot of diameters for tube-opening and lobe-widening circles and **(B)** histogram of the asymmetry angle.

| 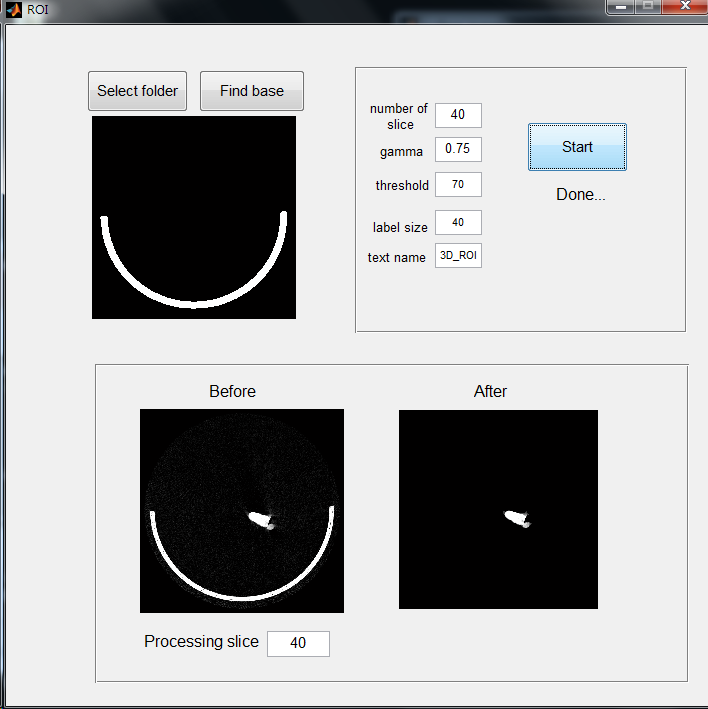 |
| --- |
| **Supplementary figure 1**: The screenshot of the flower segmentation and quality improvement program. |

| 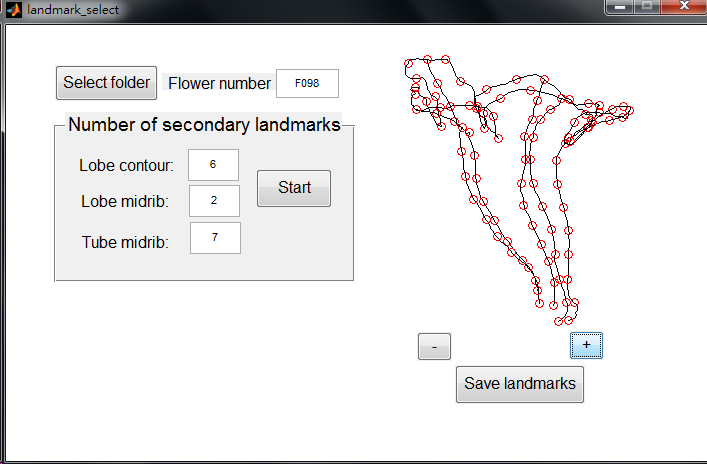 |
| --- |
| **Supplementary figure 2**: The screenshot of the landmark identification program. |

**
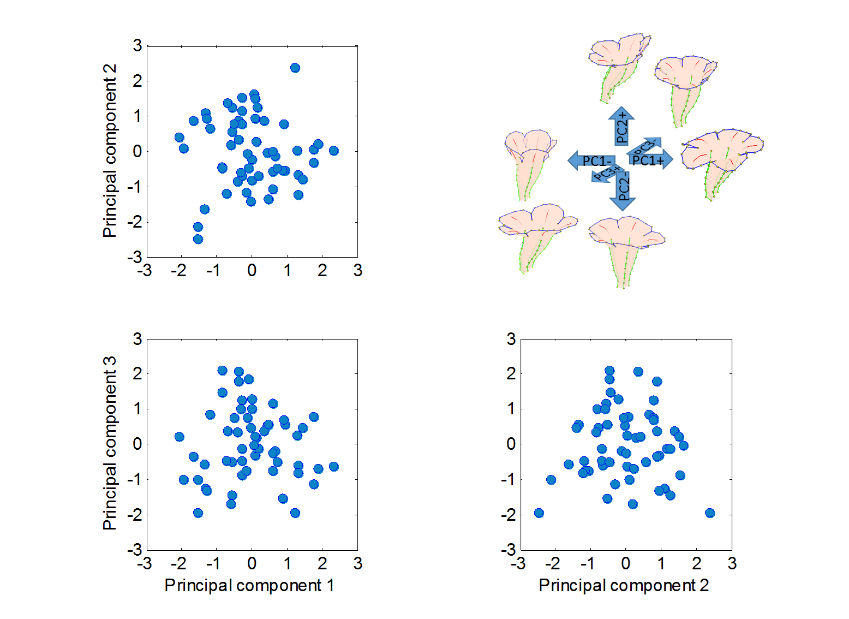
**

**Supplementary figure 3**: Scatter plots of the first three principal components.

|  |  |
| --- | --- |
| **(A)** | **(B)** |

**Supplementary figure 4**: **(A)** Boxplot of diameters for tube-opening and lobe-widening circles and **(B)** histogram of the asymmetry angle.
